# The Effect of Cerebral Palsy Disease Severity, Socioeconomic Status, and Educational Background on Selective Percutaneous Myofascial Lengthening Reoperation Rates

**DOI:** 10.7759/cureus.7336

**Published:** 2020-03-20

**Authors:** Mehdi Faraji, David Yngve

**Affiliations:** 1 Radiology, Louisiana State University Health Sciences Center, Shreveport, USA; 2 Orthopaedic Surgery and Rehabilitation, The University of Texas Medical Branch, Galveston, USA

**Keywords:** cerebral palsy, spml, reoperation, socioeconomic, education

## Abstract

Cerebral palsy (CP) is a neuro-developmental disorder. Spastic CP is the most common type of CP and is characterized by contractures of the extremities. Selective Percutaneous Myofascial Lengthening (SPML) is a minimally invasive procedure practiced by a handful of physicians in the US, and it decreases contractures and increases the range of motion in individuals with spastic CP. This study wanted to examine if there was an association between CP severity, socioeconomic status, and reoperation rates. This study used electronic medical records (EMR) to include 626 patients with spastic CP who had surgeries between January 2006 and December 2012. The zip codes from the EMR were used to determine the inflation-adjusted mean income and educational qualification (a high school education or higher) of the community via the US Census Bureau. Disease severity before the initial surgery was determined by using the functional mobility scale in the EMR to compute the Gross Motor Function Classification System (GMFCS) level. Then the data was graphed and averages were taken for the reoperation versus the no-reoperation populations, and Student's t-tests were run to determine statistical significance. The data showed that communities with higher education and income tended to reoperate more often. The higher number of reoperations in affluent communities could mean that either more affluent communities are better educated and know the benefits of bringing their children back for reoperation or that they require further education about physical therapy after the initial surgery to decrease the incidence of reoperation. This retrospective study is a level 2 study looking at the socioeconomic and educational backgrounds and disease severity and their association with reoperation rates.

## Introduction

Cerebral palsy (CP) is a group of non-progressive permanent disorders that affect movement and are attributed to non-progressive disturbances that occurred in the developing fetal or infant brain. CP can be accompanied by other secondary disturbances, such as communication difficulties [1]. Spastic CP is one of the most common types of CP. Spastic CP can be subcategorized based on the clinical picture and it usually leads to contractures that will need to be surgically released [2]. Selective Percutaneous Myofascial Lengthening (SPML) is one method that is used to release some of the contractures. SPML has many benefits such as being very quick, less invasive, and leading to faster recovery [3]. The Gross Motor Function Classification System (GMFCS) is a standardized tool that categorizes patients with CP on a scale of 1-5 with independent movement being the least at level five.

The goal of this retrospective study was to examine if there was any association between the SPML reoperation rates and the disease severity of the patients as well as their educational and socioeconomic backgrounds. The socioeconomic status of the patient may affect the outcome of SPML surgery and the need for reoperation. Socioeconomic status can also affect access to resources such as physical therapy and orthotic services and can lead to challenges with the transition to such services. A cross-sectional study in Brazil showed that as the severity of CP increases, the functional performance decreases more significantly in lower socioeconomic levels [4]. To our knowledge, this is the first study regarding children with CP who have undergone SPML surgery.

## Materials and methods

We compared the University of Texas Medical Branch (UTMB) electronic medical records (EMR) of children with SPML surgery without a reoperation and children with SPML surgery with a reoperation at UTMB. This study included 626 patients with spastic CP who had SPML surgeries between January 2006 and December 2012. Of those patients, 107 had reoperations (17%). The zip codes from addresses before the initial surgery were used to determine the inflation-adjusted mean income and educational qualification (a high school education or higher) of the community via US Census Bureau 5-year estimates for 2012 [5]. Another factor that was taken into consideration for socioeconomic status was the payer status, i.e., Medicaid/managed care or private insurance also found in the EMR. Disease severity before the initial surgery was determined by using the functional mobility scale in the EMR to get the GMFCS level. The data was then graphed and averages were taken of the different populations and Student's t-tests were run to determine statistical significance.

## Results

**Figure 1:**
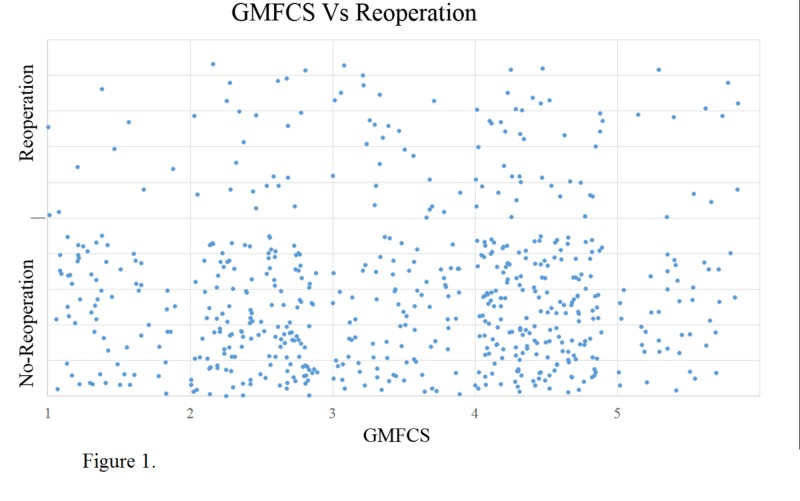
Differences in preoperation GMFCS levels between patients who received reoperation and those that did not GMFCS: Gross Motor Function Classification System

Our results showed that there was no preoperation difference in GMFCS levels in the spread of the individuals who received reoperation vs. those that did not. The mean GMFCS level of the reoperation population was 3.2 in comparison to 2.98 in the no-reoperation population; the p-value was 0.087 (Figure 1).

**Figure 2:**
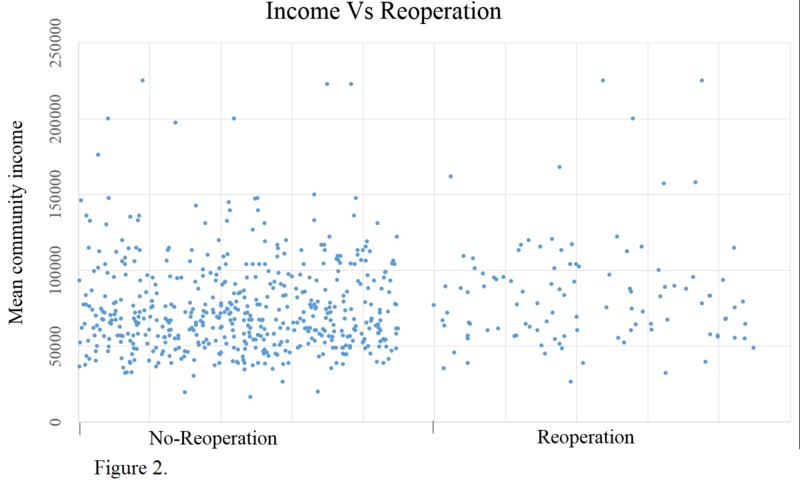
Comparison of mean community income (in US Dollars) between patients who received reoperation and those that did not

We found that the reoperation community income was higher. The mean community income of the reoperation population was USD 82,698 vs. 74,424 in the no-reoperation population; the p-value was 0.011 (Figure 2).

**Figure 3:**
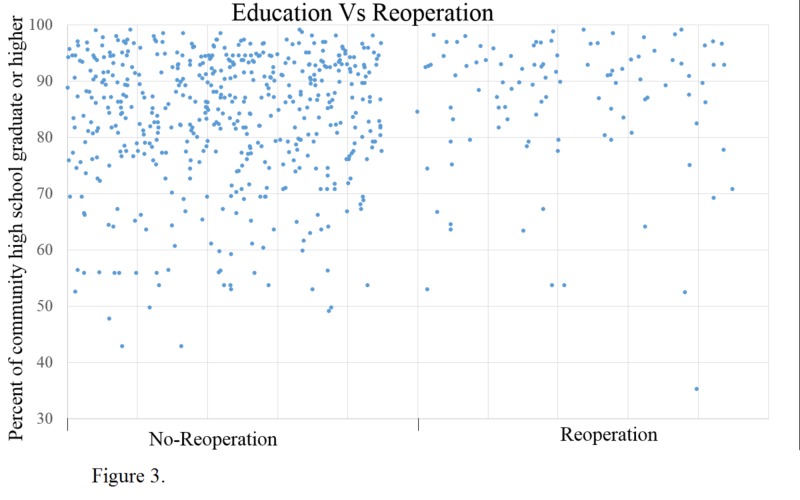
Differences in community education levels between patients who received reoperation and those that did not

The next factor we looked at was the education level of the community, and we found that the reoperation community's education level was higher. The education level was calculated as a percentage of the community that had a high school education or higher. The mean community education level of the reoperation population was 86% vs. 83.5% in the no-reoperation population; the p-value was 0.038 (Figure 3).

**Figure 4:**
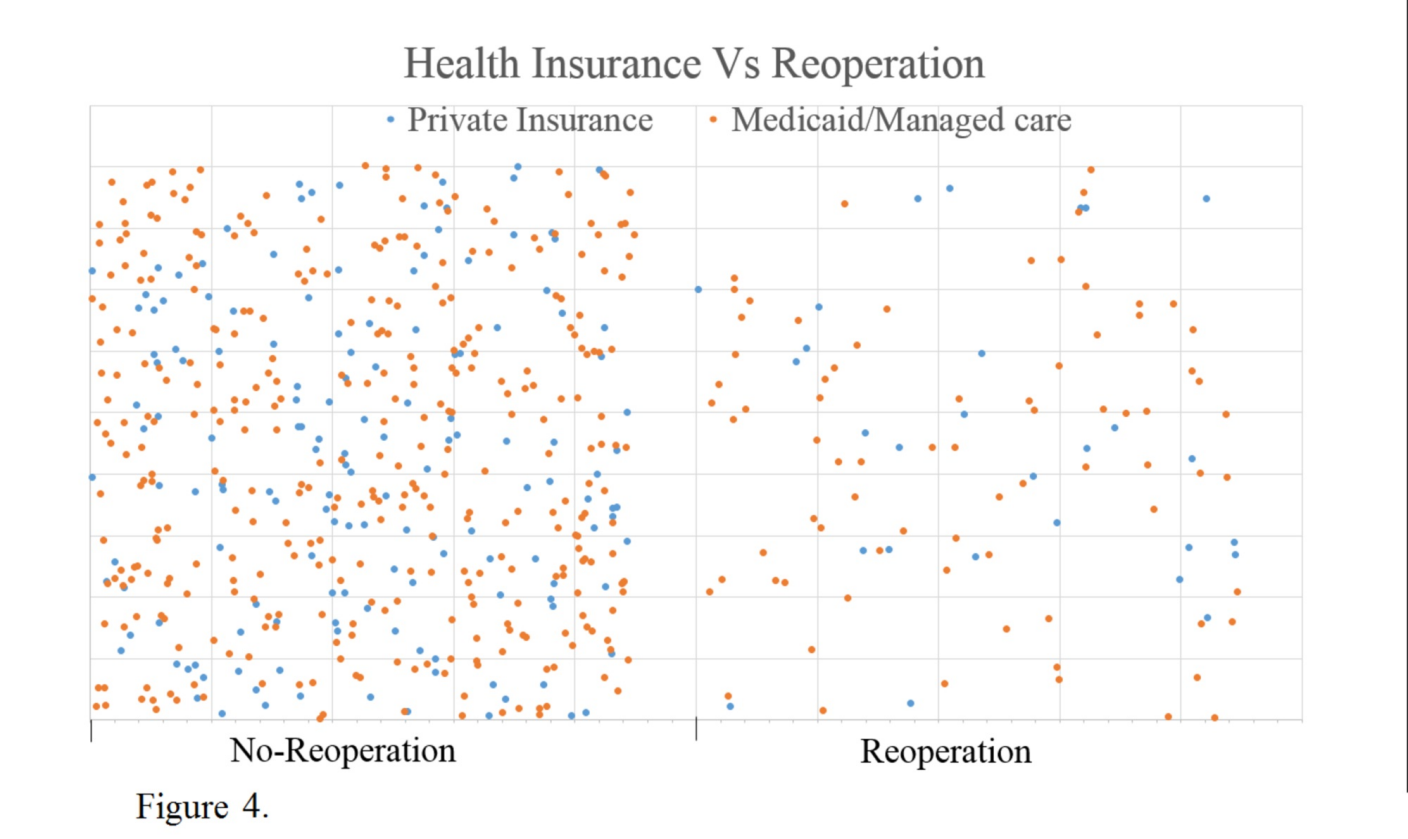
Differences in health insurance types between patients who received reoperation and those that did not

Lastly, looking at the reoperation patient insurance type, we found that the group's insurance consisted of 74% Medicaid/managed care vs. 26% private insurance. The no-reoperation population had 70% Medicaid/managed care vs. 30% private insurance. Thus, there was no significant difference in the insurance type of the reoperation group compared to the no-reoperation group (Figure 4).

## Discussion

CP is one of the most common neurological disorders that is encountered in a healthcare setting. Spastic CP can lead to contractures that can limit the quality of life in patients [6]. These contractures can be released with surgery, and one such surgery is the SPML operation. Unfortunately, sometimes healthcare providers have to go back and repeat the surgery [3]. Here we tried to look at some socioeconomic factors and how they were different in the population of patients that did and did not receive reoperations for SPML.

If socioeconomic factors decrease the quality of the result of the initial SPML operation, efforts should be made in the future to minimize the negative effects of those factors. This study showed no significant difference between the mean initial preoperation GMFCS value in the reoperation and no-reoperation populations. The data shows statistical significance in that people belonging to less educated and lower socioeconomic communities tend to reoperate less frequently (p-value of 0.011 for inflation-adjusted community income and 0.038 for community education of high school or higher), which suggests that if reoperation is necessary, they could benefit from further education about reoperation after their initial surgery. It could also mean that patients from lower educated and lower socioeconomic communities partake in more physical therapy and other services, decreasing the need for reoperation. It is suggested that more affluent individuals should be educated about ways that they can ensure that the patient will be less likely to need reoperation. Also, there seems to be a trend that fewer patients with private insurance receive reoperation. It is not clear whether this is an education issue or if it could be attributed to denial of coverage.

A future study could be done to look at ethnicity and community access to green space for physical therapy to see if there is a correlation between these factors and reoperation rates. This study was retrospective and has limitations, such as relying on available documentation in the EMR by different providers. Also, the two groups that we studied were not of the same size in terms of the number of individuals, and the follow-up was limited to our chart-search period.

## Conclusions

SPML can significantly increase the quality of life in CP patients and increase their range of motion, but it may sometimes require reoperation. Our data demonstrated that more affluent communities tended to have a higher incidence of SPML reoperations. The higher number of reoperations in affluent communities could mean that either more affluent communities are better educated and know the benefits of bringing their kids back for reoperation or they require further education about physical therapy after the initial surgery to decrease the incidence of reoperation.
